# The Implementation Outcomes and Population Impact of a Statewide IT Deployment for Family Caregivers: Mixed Methods Study

**DOI:** 10.2196/63355

**Published:** 2024-12-10

**Authors:** Orly Tonkikh, Heather M Young, Janice F Bell, Jessica Famula, Robin Whitney, Jennifer Mongoven, Kathleen Kelly

**Affiliations:** 1 Cheryl Spencer Department of Nursing University of Haifa Haifa Israel; 2 Betty Irene Moore School of Nursing University of California Davis Sacramento, CA United States; 3 Valley Foundation School of Nursing San Jose State University San Jose, CA United States; 4 Family Caregiver Alliance San Franciscio, CA United States

**Keywords:** web-based assessment, caregiver, technology implementation, Consolidated Framework for Implementation Research, CFIR, information technology, IT, family caregivers, eHealth

## Abstract

**Background:**

In 2022, the US Department of Health and Human Services released the first National Strategy to Support Family Caregivers, identifying actions for both government and the private sector. One of the major goals is to expand data, research, and evidence-based practices to support family caregivers. While IT tools are widely deployed in health care settings, they are rarely available at scale in community agencies. In 2019, the state of California recognized the importance of a statewide database and a platform to serve caregivers remotely by enhancing existing service supports and investing in a web-based platform, CareNav. Implementation commenced in early 2020 across all 11 California Caregiver Resource Centers.

**Objective:**

This paper describes the implementation strategies and outcomes of the statewide implementation of CareNav, a web-based platform to support family caregivers.

**Methods:**

The Consolidated Framework for Implementation Research (CFIR), including a recent addendum, guided this mixed methods evaluation. Two major approaches were used to evaluate the implementation process: in-depth qualitative interviews with key informants (n=82) and surveys of staff members (n=112) and caregivers (n=2229). We analyzed the interview transcripts using qualitative descriptive methods; subsequently, we identified subthemes and relationships among the ideas, mapping the findings to the CFIR addendum. For the surveys, we used descriptive statistics.

**Results:**

We present our findings about implementation strategies, implementation outcomes (ie, adoption, fidelity, and sustainment), and the impact on population health (organizational effectiveness and equity, as well as caregiver satisfaction, health, and well-being). The platform was fully adopted within 18 months, and the system is advancing toward sustainment through statewide collaboration. The deployment has augmented organizational effectiveness and quality, enhanced equity, and improved caregiver health and well-being.

**Conclusions:**

This study provides a use case for technological implementation across a multisite system with diverse community-based agencies. Future research can expand the understanding of the barriers and facilitators to achieving relevant outcomes and population impact.

## Introduction

### Background

Family caregiving is gaining visibility as an important public health issue, with 1 in 5 families engaged in long-term care for older adults and persons with disabilities often lacking adequate resources and supports [1]. The complexity and intensity of caregiving for older adults and persons with disabilities is increasing as the population ages, and more individuals live longer with physical, cognitive, and mental health challenges. Family caregivers enable family members and friends to live with chronic conditions in their environments of choice, assist with navigating acute health crises and hospitalizations, and provide comfort and support at the end of life. More than half of all family caregivers provide complex care, including medical or nursing tasks previously performed in inpatient settings, delivering most of the care after discharge from hospitals [2]. State-level data reveal that in California, at least 4.4 million family caregivers assist individuals aged >18 years; of these caregivers, more than half (56%) are employed while providing care. These individuals provide an estimated US $81 billion worth of unpaid care each year [3].

Caregivers remain relatively invisible in the health care system, to their employers, and in their communities; yet, they bear the brunt of delivering most of the long-term care for the aging population. Caregivers report a lack of knowledge regarding the best caregiving practices, often learn how to deliver care on their own, and are worried about making mistakes [2]. Most caregivers are employed, but their income is often compromised by the caregiving role [3]. While family caregivers report positive aspects of caregiving, they also experience strain, depression, and loneliness; moreover, they neglect their own health-related conditions because of the caregiving role [2]. There is evidence of health disparities in caregiving demands, supports, and resources among diverse populations by race or ethnicity and socioeconomic status [4]. Recent systematic reviews have concluded that caregivers require information and skills training, tools to improve coping with the physical and emotional burden of caregiving, paid and unpaid help, effective communication with the person in their care, and support to address barriers as they navigate the health care system [5,6].

In 2022, the US Department of Health and Human Services released the first National Strategy to Support Family Caregivers, identifying actions for both government and the private sector [7]. One of the major goals is to expand data, research, and evidence-based practices to support family caregivers. While IT tools are widely deployed in health care settings, they are rarely available at scale in community-based agencies. Clinical settings use consumer-facing features such as secure internet portals that enable individual access to the electronic health record and facilitate secure email messaging between the person and the health care provider, as well as internet-based resources for education, information, advice, and peer support [8]. IT tools could be highly beneficial for community-based caregiver assessment, service delivery, and the evaluation of a broad range of interventions, increasing access and convenience. To date, most projects examining innovative IT tools focus on feasibility and acceptance with studies of limited sample sizes [9-11].

### The California Caregiver Resource Center System

Established in 1984 by the Comprehensive Act for Families and Caregivers of Brain-Impaired Adults to support caregivers and care recipients, the California Caregiver Resource Center (CRC) system includes 11 sites with catchment areas covering the entire state. Staffed with administrators, family consultants, and educators, the CRCs respond to caregiver inquiries or referrals by conducting a short intake followed by a structured standardized assessment should the caregiver want to proceed. Before 2020, these assessments were completed on paper, either in person or by telephone. In 2020, the CRCs conducted 6126 intakes, with 4299 proceeding to full assessment. In some cases, caregivers have a simple query that is satisfied with the initial contact; others seek longer-term support. After the assessment, family consultants develop recommendations for community-based services and supports based on caregiver priorities and needs, including appropriate referrals for respite, counseling, financial or legal consultation, education, and support services. The CRCs do not address the health care needs of the care recipient; instead, they recommend follow-up with health care providers.

### The Implementation of a Statewide Caregiver Database

Recognizing the importance of a statewide database and a platform to serve caregivers virtually, in 2019, the California Department of Health and Community Services invested in a web-based platform, CareNav, across the existing support network, the California CRC system. The Department of Health and Community Services committed to funding expanded caregiver services and the deployment of CareNav over 3 years (2019-2022). In essence, the deployment of CareNav converted a manual record system to an electronic system with expanded functionality to manage client services and supports. The proprietary software platform CareNav was developed by the San Francisco Bay Area CRC, Family Caregiver Alliance, in collaboration with software developer Quality Process [12]. CareNav enables standardized caregiver self-assessment, a web-based record of client information and encounters, secure communications, and the ability to create a care plan as well as tailored information and resource content. Clients may complete the assessment either on the web or by contacting staff members who administer and document the assessment in the electronic record. The system includes administrative functionality for tracking service authorizations and contracts, generating aggregate profiles, and producing management reports on staff activities.

Led by the Family Caregiver Alliance, statewide CareNav implementation included related training for all CRC sites on platform use, data quality improvement, and change management. The University of California Davis Family Caregiving Institute was engaged to evaluate the implementation process. A previous publication described the implementation process and early outcomes [13]. The Consolidated Framework for Implementation Research (CFIR) guided our evaluation [14]. Early findings indicated that leadership, communication, the harmonization of processes across sites, and motivation to serve clients using technology were critical elements of success.

**Figure 1:**
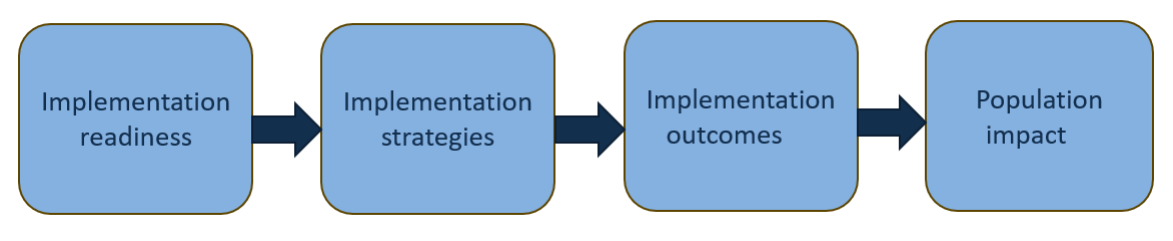
Consolidated Framework for Implementation Research addendum.

The aim of this paper is to describe further progress, including implementation readiness, implementation strategies, and subsequent implementation outcomes and population impact of the web-based resource IT tool or platform (CareNav) at the 11 California CRCs. A recent addendum to the CFIR went beyond evaluating the outer setting, the inner setting, the intervention characteristics, staff characteristics, and the process of implementation by augmenting the original model with implementation outcomes and population impact [15]. The dynamic sustainability framework [16] informed the interpretation of the sustainability of implementation outcomes. The conceptual model for this study incorporated both the CFIR addendum and the dynamic sustainability framework, adapted from the work of Damschroder et al [15] and Chambers et al [16], and is depicted in Figure 1.

## Methods

We used a mixed methods sequential triangulation design to examine the implementation process and outcomes, including focused interviews with CRC staff members and leaders and surveys of both staff members and caregiver clients.

### Ethical Considerations

The study was determined as exempt research and approved by the University of California Davis Institutional Review Board (IRB ID: 1561379-2). No identifying information was collected from participants in the focused interviews and surveys. Participants were informed of the study’s purpose and the voluntary nature of participation. In the focus interviews, participants provided oral consent to participate and to be recorded. In the surveys, participants provided assent by completing the survey.

### Recruitment

We recruited participants from the 11 California CRC sites. For focused interviews, we sent an email invitation to all leaders and clinical staff members as well as the implementation team. All current leaders and staff members of the 11 CRCs were eligible to participate in the focus group interviews. For the staff survey, we sent email invitations to all CRC staff members to complete. The survey was administered electronically between July and September 2023. For caregiver surveys, the sites distributed surveys quarterly to all caregivers served in the past quarter (4 quarters during the year between July 2022 and June 2023). Caregivers were given the option to complete the survey electronically or on paper, mailing the response to the sites, whose staff members entered the data in an electronic database.

### Focused Interviews

We conducted within-site focus group interviews with leaders and staff members of each site (22 focus groups in total) and interviewed 2 key informants from the implementation team. After obtaining consent to participate and be recorded, we used a semistructured interview guide to explore the CareNav implementation process, challenges and facilitators, anticipated system and client outcomes, satisfaction with the process, and training activities. The interviews were conducted over Zoom (Zoom Video Communications, Inc) in March and April 2022 and audio recorded. The duration of the interviews ranged from 45 to 60 minutes.

### Surveys

#### The Readiness Survey

The readiness survey is a tool assessing staff preparation and confidence regarding the implementation process and self-efficacy using a 5-point scale (1 represents the most negative response and 5 the most positive response) [13]. The readiness survey also assessed knowledge about CareNav, caregiver support to use CareNav, and implementation outcomes encompassing CareNav adoption and developmental phases (fidelity). Six items addressing developmental phases rated current CareNav use and willingness to expand CareNav use according to functionalities previously identified in the focused interviews. These items were tailored to either clinical or administrative staff, depending on role. Clinical staff responded to 15 items focused on using CareNav to guide assessment and encouraging clients to access CareNav. Administrative support staff responded to 14 items focused on using CareNav data to make decisions. Open-ended questions identified benefits and concerns about CareNav, as well as suggestions for improvement.

#### The Caregiver Satisfaction Survey

The caregiver satisfaction survey assessed satisfaction with services, confidence in caregiving, knowledge, caregiver stress, and experiences with the web-based platform and technology. The surveys included items rated on a 5-point scale. Scores range from 1 (*strongly disagree*) to 5 (*strongly agree*), with 5 indicating the most positive impact. The survey also invited comments from caregivers in an open-ended format. Surveys were translated into Spanish and back translated into English and clients could select either English or Spanish versions.

To encourage caregivers to participate in the satisfaction survey in a safe environment and minimize social desirability bias, the surveys were anonymous, and associated demographic data were not collected.

### Data Analysis

The recordings of the focus group interviews were transcribed, audited, and then imported into Dedoose qualitative data analysis software (SocioCultural Research Consultants, LLC). We used qualitative descriptive methods to analyze the transcripts and open-ended responses to the surveys [17,18] and established a 3-phase protocol for analysis. In the first phase, 3 members of the research team reviewed the transcripts and developed initial codes and definitions. In the second phase, 2 team members coded the transcripts, meeting weekly with the third member to discuss coding decisions; refine code definitions; reach consensus about the coding; and identify themes, subthemes, and relationships among the ideas. In the third phase, we mapped the themes to the CFIR addendum and the dynamic sustainability framework. We documented analysis notes, codes, and refinements in an audit trail.

Quantitative data from both surveys were analyzed using SPSS software (version 27.0; IBM Corp) to generate descriptive statistics. Mixed methods data analysis was performed after the completion of separate analyses of the survey and focused interview data. During this phase, we integrated quantitative and qualitative results and interpreted the findings in relation to the CFIR addendum and the dynamic sustainability framework.

## Results

### Participants

Across 11 CRC sites, 80 members of the staff (clinical staff members [family consultants or social workers]: n=43, 54%; administrative support staff members: n=10, 13%; and leaders [directors, clinical directors, and managers]: n=27, 34%) participated in 22 site-specific focus group interviews, with between 2 (2%) and 15 (19%) participants per site. In addition, we conducted 2 individual interviews with key informants from the implementation team.

For the readiness survey, there were responses from 118 staff members, of whom 112 (94.9%) completed at least 80% (12/15 for clinical staff and 13/14) of the readiness survey responses, and 105 (89%) submitted a demographic survey. Most of the participating staff members served in clinical roles (70/112, 62.5%), and the remaining (42/112, 37.5%) were in administrative roles.

All samples were diverse across age and racial identity, with most of the participants being female (60/82, 73% in the focused interviews and 85/105, 80.9% in the readiness survey). Demographic characteristics of the samples are presented in Table 1.

For the caregiver satisfaction survey, of the 5782 caregivers served during fiscal year 2022-2023, a total of 2229 (38.55%) responded. In addition to responding to the survey items, caregivers were invited to make comments about the services. Caregivers provided 1210 comments about the services, of which 40 (3.31%) were in Spanish. While we did not collect demographic data in these anonymous surveys, we present the characteristics of the population of caregivers who completed assessments between July 2022 and June 2023 with the CRC sites (n=5782) in Multimedia Appendix 1.

We present survey results and major themes from the qualitative data that reflect major components of our conceptual model: implementation readiness, implementation strategies, implementation outcomes, and population impact.

**Table 1:** Demographic characteristics of staff members participating in the focused interviews and readiness survey.

Participant characteristics			Interviews (n=82), n (%)		Readiness survey (n=105)^a^, n (%)
**Age (y)**					
	≤25	8 (9.8)		1 (1)	
	26-35	30 (36.6)		39 (37.1)	
	36-45	11 (13.4)		27 (25.7)	
	46-55	12 (14.6)		11 (10.5)	
	56-65	11 (13.4)		13 (12.4)	
	>65	4 (4.9)		6 (5.7)	
	Declined to answer	6 (7.3)		8 (7.6)	
**Sex**					
	Female	60 (73.2)		85 (81)	
	Male	16 (19.5)		13 (12.4)	
	Other	1 (1.2)		3 (2.9)	
	Declined to answer	5 (6.1)		4 (3.8)	
**Racial and ethnicity^b^**					
	African American or Black	4 (4.9)		5 (4.8)	
	Asian or Pacific Islander	12 (14.6)		17 (16.2)	
	Hispanic or Latinx	34 (41.5)		39 (37.1)	
	Native American	2 (2.4)		2 (1.9)	
	White	30 (36.6)		37 (35.2)	
	Other	1 (1.2)		2 (1.9)	
	Declined to answer	5 (6.1)		10 (9.6)	

^a^Of the 118 survey respondents, 105 (89%) submitted demographic surveys.

^b^Percentages may not total 100 because respondents could select multiple racial identities.

### Implementation Readiness

We assessed the implementation readiness of staff members with the readiness survey. Overall, participants had very positive attitudes toward the implementation of CareNav, with a mean total readiness score of 4.3 (SD 0.5) on a scale of 1 to 5, where 5 represents the most positive response. Average responses to all items were in the positive range (Table 2).

**Table 2:** Readiness survey responses 3 years after implementation launch (n=112).

Item			Score, mean (SD)
**Knowledge and beliefs about CareNav**			
	CareNav improves the ability to record services		4.4 (0.8)
	CareNav provides tailored and accessible information for caregivers		3.8 (1.0)^a^
	Clients should be given a range of service delivery options to ensure they select one that works best for them		4.8 (0.5)
**Self-efficacy**			
	Prepared to use CareNav		4.3 (0.7)^a^
	Confident to use CareNav		4.4 (0.7)
	Capable to use CareNav		4.5 (0.7)^b^
**Readiness for change**			
	Positive regarding the expansion of CRC^c^ services		4.4 (0.8)^b^
	Positive regarding using CareNav		4.1 (0.9)^a^
	Willing to do new things		4.4 (0.7)
	Know where to obtain help		3.5 (1.3)
	Total readiness score		4.3 (0.5)
**Developmental phases of CareNav implementation**			
	Use CareNav data to understand the needs of diverse clients		3.7 (1.3)
	**Clinical support**		
		Use CareNav to guide assessments and enter data in real time	4.3 (1.0)
		Encourage clients to access CareNav through the portal	3.1 (1.2)
		Would like to expand use of CareNav to coordinate client support	3.8 (1.0)
	**Administration**		
		Use CareNav data to make decisions regarding the CRC site and its programs	3.6 (1.0)
		Would like to expand use of CareNav	4.3 (1.0)

^a^n=111.

^b^n=109.

^c^CRC: Caregiver Resource Center.

### Implementation Strategies

#### Overview

**Figure 2:**
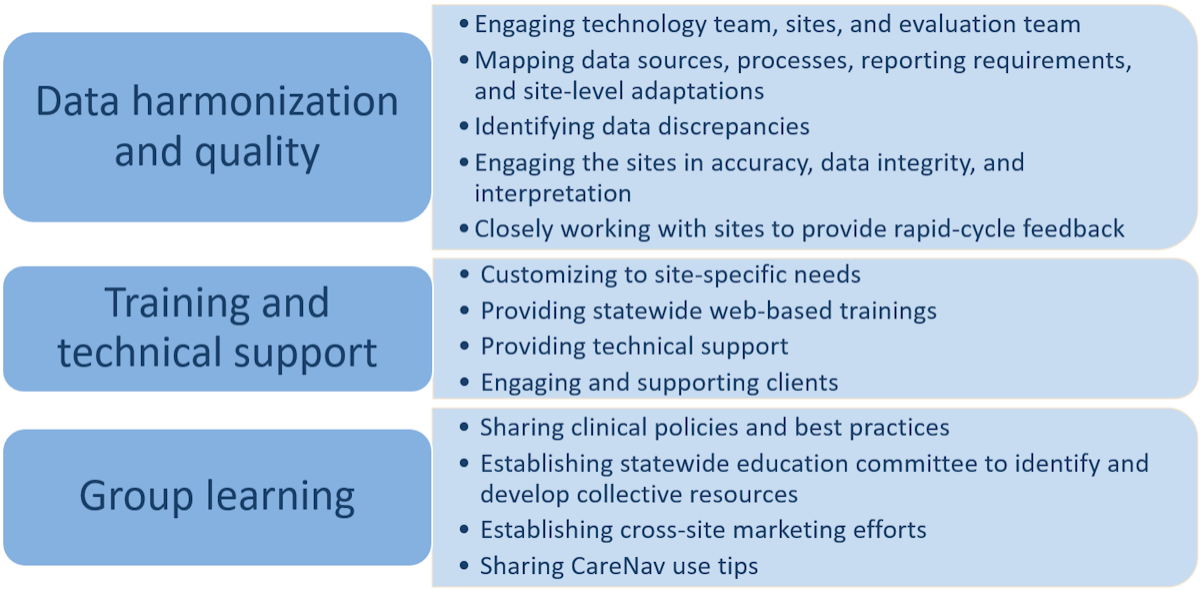
Implementation strategies.

The implementation process entailed iterative, continuous, and long-term activities using real-time analysis of client data with adaptation and refinement. Focus group participants and key informants described 3 main strategies used: data harmonization and quality, training and technical support, and group learning. Figure 2 summarizes the implementation strategies.

#### Data Harmonization and Quality

The goal of creating a statewide database and service management system carried several important assumptions, including standardizing the assessment, adopting common workflows around services and referrals, and agreeing to shared metrics for success. CRC site staff members and the Quality Process technology team engaged in data harmonization and quality, facilitated by the evaluation team. During the early part of the project, the design team engaged in deep learning at each site to understand the local conditions and to map the technology implementation path. The overall approach to initial deployment was to optimize the common elements and to minimize customization. The philosophy of designing and scaling CareNav necessitated balancing the unique data collection and integration needs of each site, with the goal of creating a state-level decision support and resource provision system to expand services for California caregivers. Thoughtful decisions were made regarding the extent of the site-level flexibility that the system could support for each CareNav feature without compromising uniformity.

The evaluation team conducted extensive analysis of CareNav data, including variables collected at intake, assessment, and reassessment, providing rapid-cycle feedback to the CRC sites when data discrepancies were identified. The evaluation team also raised issues around data quality and integrity to the CRC directors and clinical directors for discussion and consensus building. Implementation involved data harmonization—the integration of data sources and structures from the 11 sites—requiring processes to ensure data quality and consistency. There were 2 major issues in the implementation of a standardized assessment: mapping previous data to the appropriate fields in CareNav and coming to consensus on variable definitions to reconcile diverse interpretations of specific data points.

Several threats to data quality had to be resolved through consensus building. First, staff members held diverse beliefs about data accuracy and quality, which led to different practices in collection and entry; for example, staff members varied in how they conducted and recorded assessments, ranging from the majority of consultants administering the standardized assessment and completing data fields in a systematic way into the electronic record to consultants at 1 site using the standardized assessment as a general guide for conversation and later entering their interpretation of the client’s narrative as data. At this site, consultants conducted guided interviews, after which another staff member entered the data. Data integrity was further threatened because staff members interpreted the meaning of data fields differently and subsequently recorded with this bias. These issues were compounded when staff members conducted the interviews in languages other than English (the only language currently supported by CareNav), then translated and entered data. Complex concepts, such as spirituality or loneliness, carry different cultural meanings and are subject to linguistic inaccuracy across translation. Finally, site leaders varied in their commitment to ensuring data quality and in their ability to provide guidance to their teams to achieve consensus and accuracy.

#### Training and Technical Support

Technology deployment requires extensive training and technical support. Users begin with different levels of technical and data understanding; therefore, leaders must customize training to establish shared foundational knowledge and skills and to address site- and person-specific learning needs. Furthermore, CareNav features a client portal, necessitating the preparation of caregivers—both in general digital access and specific coaching—to use the program. The implementation team advanced training and support in several ways, with site-specific education sessions, statewide web-based training, and site and individual technical support, as well as assisting staff members to support caregivers [13].

#### Group Learning

The statewide deployment of CareNav offered the CRC sites new opportunities for collaboration and group learning. Several subgroups were formed, including those focused on clinical, leadership, and data management issues. Within these subgroups, members across the state engaged with one another in lessons learned and shared educational materials and best practices among the sites. A statewide education committee was formed to identify and develop collective resources for both staff members and clients. The collective also focused on cross-site marketing efforts, including a central website with links to all sites, enhancing the general visibility of caregiver resources and improving the ability of clients to find the appropriate support.

### Implementation Outcomes

**Figure 3:**
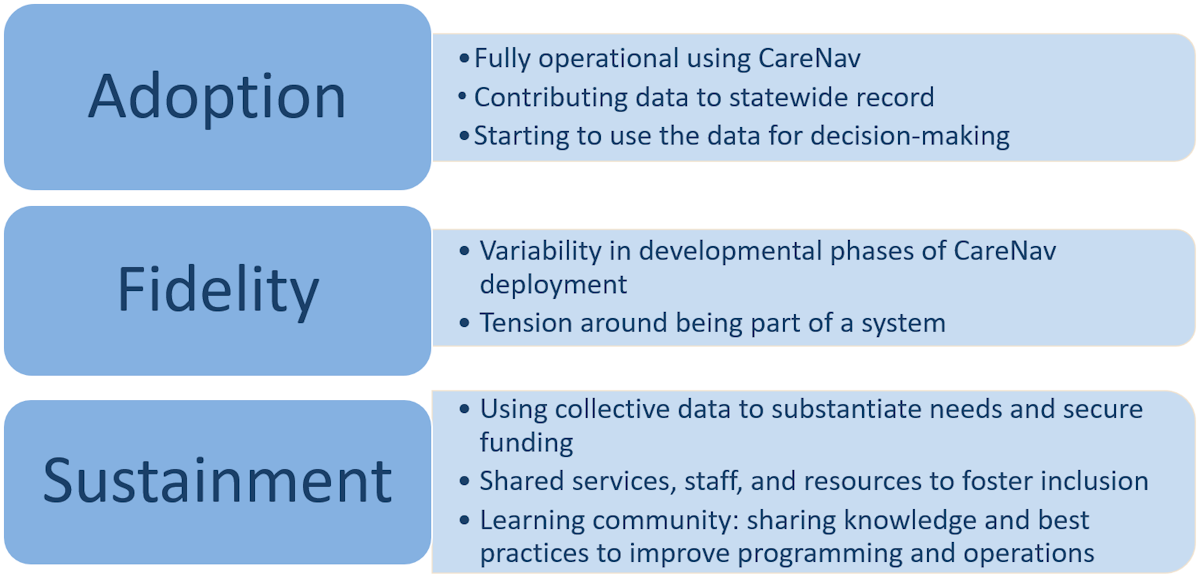
Implementation outcomes.

Implementation outcomes (adoption, fidelity, and sustainment) were assessed using qualitative and survey data gathered from staff members and clients. Figure 3 summarizes the main themes encompassing implementation outcomes.

#### Adoption

By fiscal year 2021-2022 (approximately 2 years after launching CareNav at all California CRC sites), all CRCs had adopted the new platform and were fully operational, using CareNav and contributing data to the statewide record. A year later, in 2023, most of the staff survey participants (101/112, 90.1%) agreed (somewhat or strongly) that everyone on staff regularly used CareNav.

#### CareNav Functionality

Participants described the most significant features of CareNav: the standardized assessment, reports, and the client portal. The CRC sites use these CareNav features for 2 primary purposes: as client records to facilitate serving individual clients and for consultant- or site-level management of the client population. Table 3 summarizes CareNav features and their use, along with more detailed description.

**Table 3:** Standardized assessment, report generation and a client portal are CareNav features.

Functionality		Standardized assessment	Report generation	Client portal
**Client level**				
	Client records	Data collection and service history	Client use and web-based resource use	Self-administration of intake and assessment
	Case management and decision support	Access to client records for all staff members on the team	Aggregate client data and units of service	Messaging clients and assigning resources
	Staff-client interaction and service provision	Generating tailored resources	Client engagement and units of service	Asynchronous access and communication
**Consultant, site, and system levels**				
	Caseload management to support efficiency	Navigation features (search, sort, and filter)	Clinical staff caseload	—^a^
	Outreach and diversity, equity, and inclusion	Ease of access for diverse clients	Outreach and service for target populations	Convenient access to services
	State-level planning	Aggregate summary of client needs	Populations served and service provision	—

^a^Not applicable.

The most common and universal use of CareNav is for client data collection. The sites described the advantages of having streamlined longitudinal records that are accessible in real time. CareNav provides ready access across staff, facilitating the continuity of client service and support, as well as case management along the service trajectory. CareNav improves team capability, facilitating care coordination among staff members, streamlining client communication, and building trust. Clinical directors and administrators use aggregate client data to manage and assign caseloads for staff members and guide outreach and program planning priorities. At the system level, standardized data collection has enabled the first-ever comprehensive view of caregiver needs in California, populations served, a comparison of clients to other caregivers in state or national databases, and service provision. CRC directors identified the power of this information to guide strategy, resource allocation, and policy:

One of the things I love as a director, is how we can look at the data and what we can do with the data...I’m able to look at our numbers and look at sort of our demographics, look at the profile of who we’re serving, and I’ve been able to take that information and talk to county funders about...who are the caregivers in...County that your funding has to serve?...We were able...to look at the difference between what’s going on with our folks in rural areas and what’s going on in nonrural areas. And it was a surprise to people that, it’s the same... And that was really awesome, because we were expecting our rural folks to be faring probably, I think worse because they were more isolated...So we can use that internally as well...And we looked at...our need to be more accessible to our diverse populations...I was able to...look at...some of our rural areas, and...we haven’t really served our Indian population, our tribal communities, as well as I think that we should.

Director

The client portal is a major innovation for CRC service delivery, providing clients with continuous access to the CRCs to post queries, complete assessments, review tailored resources, receive service vouchers, and communicate with CRC clinical staff members asynchronously. The adoption of this feature is a multifaceted process that requires the involvement of clinical staff members and clients. The clinical staff members reported that some clients prefer self-administration of the intake and assessment because it is faster and more convenient than an interview, while others prefer engaging with staff members for the assessment. CRC clients reported that they appreciate the convenience of choosing a time to complete the assessment, the privacy compared to speaking on the telephone, and the ability to complete part of the assessment and return to it later:

We appreciate having the access to be able to do the assessment and the reassessment online and to be able to compare especially...an old reassessment to what we’re doing now, so you can see side by side...so I can see the changes and that helps generate a little bit more conversation...if we see a bigger change...we can have that conversation of what happened, how has that impacted you as a caregiver? And...being able to offer a little bit different resources, then maybe...I wouldn’t have been able to do before, just because I wasn’t able to see the change.

Staff

The client satisfaction survey collected information about caregiver experiences with the web-based platform. Of the 2125 respondents, most were offered web-based services (n=1734, 82%), and a quarter of the respondents (n=533, 25%) indicated having used the CareNav system. Most of the caregivers who used CareNav were satisfied with the experience (extremely satisfied: 255/533, 47.8%; somewhat satisfied: 170/533, 31.9%). Those who did not use CareNav were asked about the reasons for not engaging with the web-based program. The largest barrier to use was awareness about the program (439/1390, 31.58%), followed by the impression that the caregiver did not need CareNav (265/1390, 19.06%) and a lack of technology experience (192/1390, 13.81%). Access to the internet (55/1390, 3.96%) and finding the platform too confusing (21/1390, 1.51%) were minimal barriers.

Staff members at sites offer varied levels of expertise and motivation in supporting clients, with some unable or unwilling to provide the necessary technological support and education to enable client self-administration. Some sites took a proactive approach, having a dedicated staff member to monitor clients who start a record and to reach out to offer support, as well as to send invitations to sign up and complete the web-based intake form before an appointment with a family consultant. A third of the clinical support staff members (23/70, 33%) in the survey reported encouraging clients to access CareNav through the portal.

#### Fidelity

Site-level analysis of CareNav use revealed that while all CRC sites now use CareNav for daily operations, individual sites represent different dynamic stages of the operational integration of CareNav, outreach approaches, and expansion of services, as well as diversity, equity, and inclusion efforts. The sites were categorized as *early phase* or *advanced phase* for each of the 6 dimensions. We categorized sites as *early phase* if they focused on more technical, basic, and passive actions and as *advanced phase* when they presented a more strategic approach and more use of data- and outcome-oriented operations. Table 4 depicts the developmental phases of the implementation of the platform across the 11 sites.

**Table 4:** Fidelity: the developmental phases of implementation by site.

Dimension	Developmental phase description		Site-level developmental phase (sites: N=11)											
	EP^a^	AP^b^	1	2	3	4	5	6	7	8	9	10	11	AP sites, n (%)
CareNav functionality	Client level: data collection and documentation	Client level: case management and decision support; consultant, site, and system levels: caseload management	EP	AP	AP	AP	AP	AP	AP	AP	AP	AP	AP	10 (91)
CareNav feature: standardized assessment	Asynchronous and selected fields; dedicated technical staff members entering paper data	Synchronous, comprehensive data collection; staff members or clients enter electronic data in real time	EP	AP	EP	EP	AP	AP	AP	EP	AP	EP	AP	6 (55)
CareNav feature: report generation	Predefined templates for reports	Flexible reports run by sites as needed; use reports for decision support	EP	AP	EP	AP	AP	AP	AP	EP	EP	AP	EP	6 (55)
CareNav feature: client portal	Passive approach	Active approach: staff members encourage and support clients to use client portal	EP	AP	AP	AP	EP	EP	EP	AP	AP	EP	AP	6 (55)
Outreach approach	Historical relationships and referral sources	Using data to guide and evaluate selective outreach	EP	AP	EP	EP	EP	EP	EP	EP	AP	EP	AP	3 (27)
Diversity, equity, and inclusion	Translation of materials and focusing on specific local ethnic groups	Broad definition of diversity (race and ethnicity, geography, LGBTQ^c^ identities, and income) and using data to identify opportunities for inclusion	EP	AP	EP	EP	AP	EP	EP	AP	AP	AP	EP	5 (45)

^a^EP: early phase.

^b^AP: advanced phase.

^c^LGBTQ: lesbian, gay, bisexual, transgender, and queer.

As can be seen in Table 4, the sites are in different phases of implementation depending on the dimension: of the 11 sites, 1 (9%) is operating at an advanced level across all dimensions, and 1 (9%) is at an early level across all dimensions. The most advanced dimension across sites is data collection and documentation, while the dimension with the lowest advancement, with only 3 (27%) of the 11 sites at advanced level, involves using data to create strategies for outreach:

Learning a new system. It just requires...time and patience and flexibility. And one thing came up...about CareNav in particular...there’s a lot of functionality built into it. We can do a lot of things with CareNav. And so right now we’re doing, maybe we’re only using a certain percentage of all of the tools that are built into it, and really learning how.

Staff

**Figure 4:**
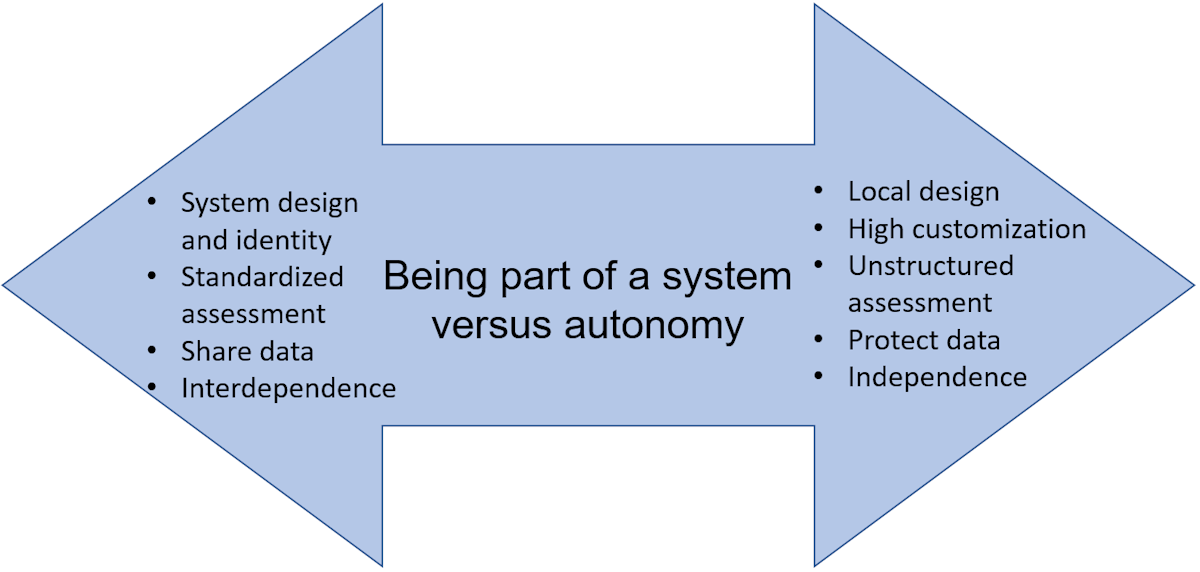
The tension between being part of a system and being autonomous.

The developmental phases were shaped by the baseline systems in place at each site and their unique local conditions and relationships. A major interview theme related to the developmental phase was how each site weighed the benefits of incorporation into the CRC system alongside the benefits of autonomy as an individual CRC (Figure 4). For some sites, CareNav implementation involved a shift in service philosophy, in addition to implementing new technology; for example, this shift triggered a tension between a professional philosophy valuing open-ended interviewing and the standardization of the intake and assessment processes, an important feature of incorporation into a larger system with a uniform database. Most of the tension around being part of a system centered on standardized assessment, coupled with the ability to customize reports to meet local needs. This tension was most acute when a site had a previous data management system in place, requiring adaptation and harmonization. Another area of tension related to the extent to which sites are proactive in reaching the population of the region versus being more reactive and relying on established referral sources. As a statewide system with a commitment to expand services, advanced sites are using data to identify unmet needs in the region and designing strategies to connect with underresourced communities and to tailor programs to meet cultural and linguistic requirements. Furthermore, they are collaborating with one another to leverage resources across regions.

The readiness survey results (Table 2) illustrate the overall extent to which CRC staff members and leaders integrated CareNav and service expansion dimensions into their operations and future plans. The highest-rated items (mean 4.3, SD 1.0) were using CareNav to guide assessments and enter data in real time and a desire to expand the use of the platform. The lowest-rated item was encouraging clients to use the portal (mean 3.1, SD 1.2). Most of the participants with clinical support roles (56/70, 80%) used CareNav to guide assessments and enter data in real time, and two-thirds (43/70, 61%) would like to expand the use of CareNav to coordinate client support. Only a third of the clinical staff members (23/70, 33%) agreed that they encourage clients to access CareNav through the portal.

A little more than half of the participants with administrative roles (23/42, 55%) used CareNav for decision-making regarding the CRC site and its programs (eg, targeted outreach and program offerings), and more than two-thirds (30/42, 71%) were willing to expand the use of CareNav (eg, generating new reports, using data for program improvement, and making decisions). Two-thirds of the participants (69/112, 61.6%) used CareNav data to understand the needs of diverse clients (eg, in terms of race, ethnicity, geography, sexual orientation, gender identity, and income). They reported using these data to implement various strategies, including collecting demographic data, targeting grant funding and outreach based on a comparison between intake data and census demographics, improving linguistic access, training staff, and expanding the resources available in CareNav.

#### Sustainment

The focused interview participants highlighted having a shared identity and mission, sharing data and collaborating as critical elements to actualize the potential of a system of delivery for California’s caregivers and to promote sustainability. The most commonly discussed system outcome was the statewide identity across the sites that has created various opportunities for current and future partnerships. Several expressed pride in being part of a system that is a model for the nation and has a goal to support all caregivers in California. Both staff members and leaders identified the creation of new structures supporting long-term collaboration between the sites and the use of the CareNav statewide comprehensive database as a path to sustainability. They recognized the power of working together and using their collective data to better serve clients and to substantiate client care needs toward the goal of securing funding for sustainability.

CRC leaders also identified system-level outcomes that benefit clients directly. They recognized the potential of shared services, staff, and resources to foster greater inclusion across race or ethnicity and language groups. This has particular impact for service to smaller or more geographically dispersed populations. The statewide shared calendar of virtual events is a prime example of the wide and efficient dissemination of useful culturally and linguistically appropriate resources across the entire state.

The CRC leaders have formed a learning community with one another, sharing best practices and knowledge to improve the quality of their programming and operations. Directors and clinical directors have developed system-wide clinical policies and engaged in cross-site marketing efforts. Several statewide meetings involving directors, clinical directors, supervisors, and education coordinators are building collective momentum. The statewide education committee enriches site-level effectiveness and creates shared resources, as a director explains:

Since we’ve gone forward with CareNav, our entire Caregiver Resource Center system has really gone through a massive enhancement. And I think a lot of it is the work that the directors and the staff have done. So we’re getting together on a regular basis. We’re meeting. We are developing, you know, policies with the clinical side, the staff, they’re getting together and they’re coming up with policies. We’ve created a marketing campaign. Through Zoom, we’re now sharing education events statewide and collecting data statewide. So again, CareNav is critical tool. But I think the driving force behind everything has been this kind of movement of the Caregiver Resource Centers coming back together, working with lobbyists, legislators, leveraging money to come in and support our efforts.

Director

### Population Impact

**Figure 5:**
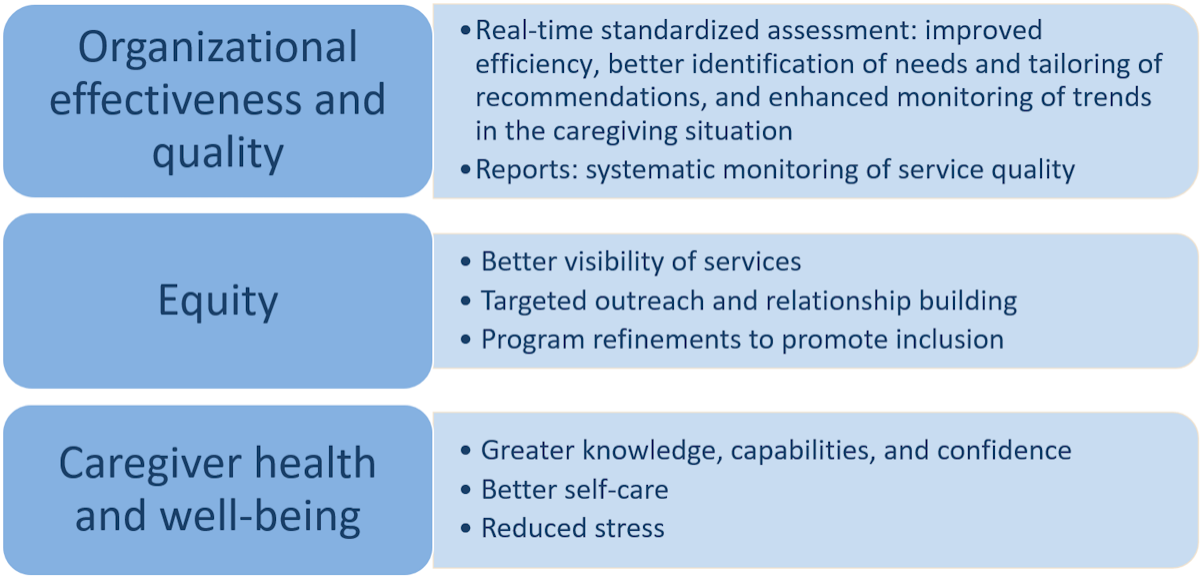
The population impact.

The impact on the population manifested in 3 major ways: achieving organizational effectiveness and quality, promoting equity, and enhancing caregiver health and well-being. Figure 5 depicts the population impact.

#### Organizational Effectiveness and Quality

Leaders described greater operational effectiveness and responsiveness as well as improved quality of services. The real-time standardized assessment assured better identification of needs and tailoring of recommendations for clients. With the documentation of repeat encounters, staff members were able to monitor the caregiving trajectory and augment resources as needed. The web-based platform enabled better communication among staff members and greater efficiency in serving clients as a team. Site leaders valued the ability to monitor the quality of service and to use data to guide decision-making around staff assignments and program priorities. The client portal facilitated timely and consistent communication with clients at convenient times for them as well as a central repository of individualized resources and services.

The most reported impact of CareNav on serving clients was the ability to provide more resources for more people in a faster and more convenient way. Many staff members noted improved client-provider relationships because CareNav allows a transparent means to provide services, accessible to both staff members and clients, fostering a more collaborative relationship. The virtual messaging tools enable timely and consistent communication. Web-based statewide resources provide more options to clients than a small regional program can offer, providing more opportunities for caregivers to access educational and support group resources from any site.

#### Equity

CareNav promotes equity by targeted subgroup analysis to better understand the experiences and priorities of diverse caregivers, enabling more thoughtful tailoring of both outreach and programs. The aggregate profiles of clients served by each CRC provided information about the reach of the programs and the gaps in service for subpopulations in the region. Synthesis of the data identified opportunities for developing new partnerships in the community to promote visibility to, and acceptability for, underserved caregivers. The data also provide guidance for strategic planning around program enhancement to achieve cultural congruence and to promote inclusion. Leaders have forged new shared services, staff, and resources to foster inclusion. A statewide shared calendar of virtual events, such as educational resources in various languages, has increased access for all regions. Several sites have collaborated to match staff members to their regional linguistic diversity, enabling the provision of consultation in the preferred language of the caregiver. Some staff members expressed concern about the digital divide disproportionately affecting certain client populations because of cost, internet access, or technological literacy. To accomplish digital equity, staff members recognize that full client engagement will require further tools and education to prepare clients to use the CareNav platform.

#### Caregiver Health and Well-Being

All parties identified benefits for caregivers, including improved health and well-being. Specifically, clients appreciated having a centralized resource that records precise identification of both care recipient and caregiver needs, coupled with tailored resources. Overall, staff members reported that CareNav has improved their ability to identify and respond to client needs and has changed the way they engage with clients:

[T]hat [the results of the assessment] gives you room to have a conversation...No wonder you’re feeling so overwhelmed. Look at, this is what you just told us. We’re not guessing you’re overwhelmed. You just told us you were overwhelmed, right, by answering these questions in that way. So, having the questions you ask in CareNav, sort of be the structure for that, the clinical interview...but taking that information and using it for developing the care plan...You said you don’t have your financial...documents in order, so...perhaps that should be on your care plan, right? Is that something that you can commit to do?...you’re feeling overwhelmed and isolated, perhaps one of our support groups might work, right?...what we ask in the assessment tells you, sort of, informs the conversation with the client.

Staff

Caregivers, staff members, and leaders highlighted the positive impact on caregiver health and well-being. The assessment followed by a tailored care plan prioritizing the most pressing concerns resulted in positive outcomes for clients. Caregivers reported gaining confidence, knowledge and awareness of community resources, better understanding of the care recipient’s situation, and better ability to manage the care they provide:

The family consultant is such a valuable resource...Helping us to connect to different resources, helping us to remember [that] we, as caregivers, shouldn’t forget to take care of our mental, emotional, and physical well-being. I am grateful they can help us to organize and make a plan to help ourselves to be there for our care receivers.

Caregiver

The caregiver satisfaction survey explored the impact of CareNav and services on the lives of the caregivers (mean scores are presented in Table 5). The results indicate strong impact in confidence and ability to manage care, increased knowledge and awareness, better access to community resources, enhanced understanding of the disease or disability and issues, improved self-care for their physical and mental health, and reduced feelings of stress.

**Table 5:** Caregiver satisfaction survey scores: the impact of CareNav and services on caregiver lives (n=2254).

Item	Score, mean (SD)
More confident as a caregiver	4.2 (0.8)
Better able to manage care	4.2 (0.8)
More knowledge and awareness	4.3 (0.8)
Understand the disease, disability, or problem better	4.1 (0.9)
Taking better care of self	4.1 (0.9)
Less stressed	3.9 (1.0)

## Discussion

### Principal Findings

This paper describes a rigorous evaluation of a complex implementation of a statewide web-based platform to enhance services provided to family caregivers. We presented findings about implementation strategies, implementation outcomes (ie, adoption, fidelity, and sustainment), and the impact on population health (ie, organizational effectiveness and equity as well as caregiver satisfaction, health, and well-being). The platform was fully adopted within 18 months, and the system is advancing toward sustainment through statewide collaboration. The deployment has augmented organizational effectiveness and quality, enhanced equity, and improved caregiver health and well-being.

The CFIR addendum and the dynamic sustainability framework provided a useful approach to explore cross-site variability and the driving forces for implementation and sustainability. A deeper consideration of outcomes can drive meaningful evaluation that includes both implementation and innovation outcomes as well as a consideration of the indicators of sustainability and impact on the population served [19].

The implementation of CareNav occurred during a unique time in history marked by rapid advances in technology in all sectors of society, changing expectations among caregivers as younger generations assumed this role, and a global pandemic. In many ways, these forces accelerated and aided the implementation process. In other ways, these collective changes deepened the divide between those who accept and embrace change and those who prefer to retain the status quo. Early implementation findings suggested that some of the sites, particularly those embedded within larger health systems, experienced a tension between harmonizing workflows with the other CRC sites and also retaining compatibility with the workflows and technologies of partner organizations [13].

Although tension around adapting individual site workflows remained a theme in this longitudinal view of implementation, site-specific technology support and training helped to address logistical barriers, and group learning provided opportunities to build consensus around which modifications were the most important. With the complexity of CRC operations, from client engagement and outreach to creating business efficiencies, it is not surprising that the sites manifest variable patterns of implementation phases across CareNav and service expansion dimensions. As has been suggested previously [20], local contextual factors drive the priority of various strategies to accomplish implementation, an observation amplified across the 11 sites in this evaluation. The variability in developmental implementation patterns is expected across a diverse network of organizations. The tendency to focus on individual client data before engaging in more advanced analytical processes provides evidence for a nonlinear implementation course of a multicomponent health IT adoption. It was helpful to the evaluation to establish the developmental phases of the implementation to consider both site-specific attainment of minimal progress and overall evolution.

The leaders of this initiative used an iterative, continuous, and long-term implementation strategy that advanced the full adoption of CareNav in daily operations. The effort was accelerated by providing appropriate training and technical support and fostering a learning community. The relatively high scores we identified across items on the readiness survey persisted over time, suggesting that these support efforts have helped to prepare CRC staff members effectively for implementation and ongoing operations.

The client adoption rates of approximately 25% exceeded the reported rates of patient portal adoption in health care systems. In a study conducted in the Netherlands, 20% of older adults who were hospitalized activated a patient portal account. The participation rate decreased with age, with approximately 50% more patients aged >76 years declining to create an account compared to those who activated one [21]. Similarly, in a community-based sample of ethnically diverse patients with low-income status attending a rural clinic in the United States, 20.5% reported using their patient portal, with greater odds of engagement for those having higher education and social support coupled with frequent internet use [22]. In the case of CareNav, the most important barrier was a lack of awareness of the opportunity to use a web-based portal. This finding contrasts with a systematic review of patient portal and electronic personal health record use, where the major barriers were privacy and security, access to the internet, and the ability to use technology [23]. Together, these findings suggest that caregiver clients are using the system at a level slightly higher than health system portals and that increasing awareness could be the most important strategy to increase engagement.

The CareNav implementation process fostered statewide system identity and created structures that had a significant role in promoting the sustainability of the implementation. Future efforts should focus on achieving sustainment and realizing population impact. Data-driven strategic decisions have the potential to realize operational efficiencies while prioritizing the most effective efforts of staff members. The system has the potential to support the documentation of population impact and cost-effectiveness as a persuasive strategy to procure sustainable funding for vital programs. Going forward, with a goal of achieving advanced implementation across all sites, training and technical support could focus more attention on enhancing site- and system-level functions and optimizing the use of data to drive both client-level and system-level decisions and priorities.

On the basis of data about the population served and gaps in program offerings, both staff members and leaders articulated the need for broader cultural adaptation of the service model for specific communities, including the linguistic translation of assessment and educational materials. Beyond language, several CRC leaders and staff members recognized the importance of a broader cultural adaptation approach for specific communities (eg, caregivers from tribal communities, underresourced racial or ethnic groups, the LGBTQ+ [lesbian, gay, bisexual, transgender, queer, and similar minority] community; and rural settings) to assure the congruence of programming with client needs and to advance equity. Several leaders cited the dearth of evidence about the best approaches to serve certain underrepresented communities and shared the hope that evaluation of their efforts will contribute to this important knowledge base.

For clients, digital equity occurs at multiple levels, from the availability of internet service in certain communities and the affordability of service to technological literacy. CRC staff members play a vital role in encouraging clients to use the web-based program and providing technical support as they navigate the system, and some of the sites are more effective at promoting web-based engagement than others. Full deployment requires overcoming these barriers and ensuring access to all caregivers who desire to participate in this way.

The limitations of this study included reliance on self-report from staff members and caregivers regarding implementation progress. This limitation was partially offset by the triangulation of actual data entered into CareNav that was analyzed by the evaluation team to identify quality and integrity issues. With the iterative process of engagement among the evaluation team and the sites implementing the program, we were able to identify progress and barriers associated with implementation in real time. The implementation occurred in a state that is more diverse demographically than many other US states, limiting generalizability to all states but providing valuable information related to equity.

### Conclusions

The study identified individual and site-level factors related to the CareNav implementation process. Future longitudinal studies should explore long-term adoption trajectories to inform continuous implementation planning, particularly to guide implementation efforts in complex health or social care systems, where one size does not fit all. Further research could examine longer-term outcomes, particularly in the areas of impact on clients served. Finally, the question of the extent to which automating social service processes and using artificial intelligence expands capacity is a vital consideration with the growth of the older adult population and the need for new solutions to increase capacity.

While electronic records are common in health systems and in a variety of industries, community-based agencies have lagged behind in adoption. Advancement in technology in this sector is essential to realize the integration of health and social services for the betterment of population health and to address the growing demand for services. The results of the efforts of the California CRCs provide a compelling use case for the successful implementation and adoption of technology in community-based agencies. Going forward, the California CRCs will grapple with important questions about being a statewide system, advancing technological capacity for clients and staff members, and solving vital equity issues to provide services and supports to all caregivers in need.

## Appendix

Multimedia Appendix 1 Characteristics of caregivers served by the California Caregiver Resource Centers (n=5782).

## Abbreviations

CFIR: Consolidated Framework for Implementation Research
CRC: Caregiver Resource Center
LGBTQ+: lesbian, gay, bisexual, transgender, queer, and similar minority
