# Supplementary material for: The Implementation Outcomes and Population Impact of a Statewide IT Deployment for Family Caregivers: Mixed Methods Study
Source: JMIR Aging. 2024 Dec 10;7:e63355. doi: 10.2196/63355 (PMC11669000; doi:10.2196/63355)
Supplement: Multimedia Appendix 1 [file aging_v7i1e63355_app1.docx]

Table S1: Characteristics of caregivers served by the Caregiver Resource Centers (n=5,782)

| Characteristic | **Percentage** |
| --- | --- |
| **Age (y)** |  |
| 18-44 | 11 |
| 45-64 | 42.2 |
| 65-84 | 43 |
| >85 | 3.8 |
| **Highest level of education** |  |
| Some high school | 2.6 |
| High school graduate | 14.3 |
| Some college | 27.9 |
| College graduate | 38 |
| Post graduate degree | 17.2 |
| **Marital status** |  |
| Married or partnered | 69.4 |
| Separated or divorced | 9.1 |
| Single | 18.5 |
| Widowed | 3 |
| **Employment status** |  |
| Full time | 29.4 |
| Part time | 10.9 |
| Retired | 41 |
| Unemployed | 15.9 |
| Leave of absence | 2.6 |
| **Caregiver lives alone** | 7.5 |
| **Caregiver lives in rural area** | 4.4 |
| **Identifies as primary caregiver** | 95 |
| **Duration of caregiving (y)** |  |
| <2 | 43.2 |
| 2-5 | 26.8 |
| >5 | 30.1 |
| **Care recipient has memory loss** | 91.5 |
| **Caregiving hours per week** |  |
| 0-10 | 6.6 |
| 11-20 | 7.4 |
| 21-39 | 12.9 |
| >40 | 73.2 |
| *Among completed assessments; deduplicated by caregiver; percentages may not add to 100 due to rounding | |
